# Lesions of the Fasciculus Retroflexus Alter Footshock-Induced cFos Expression in the Mesopontine Rostromedial Tegmental Area of Rats

**DOI:** 10.1371/journal.pone.0060678

**Published:** 2013-04-12

**Authors:** Paul Leon Brown, Paul D. Shepard

**Affiliations:** 1 Maryland Psychiatric Research Center, Department of Psychiatry, University of Maryland School of Medicine, Baltimore, Maryland, United States of America; 2 Program in Neuroscience, University of Maryland, Baltimore, Maryland, United States of America; Max-Planck-Institut für Neurobiologie, Germany

## Abstract

Midbrain dopamine neurons are an essential part of the circuitry underlying motivation and reinforcement. They are activated by rewards or reward-predicting cues and inhibited by reward omission. The lateral habenula (lHb), an epithalamic structure that forms reciprocal connections with midbrain dopamine neurons, shows the opposite response being activated by reward omission or aversive stimuli and inhibited by reward-predicting cues. It has been hypothesized that habenular input to midbrain dopamine neurons is conveyed via a feedforward inhibitory pathway involving the GABAergic mesopontine rostromedial tegmental area. Here, we show that exposing rats to low-intensity footshock (four, 0.5 mA shocks over 20 min) induces cFos expression in the rostromedial tegmental area and that this effect is prevented by lesions of the fasciculus retroflexus, the principal output pathway of the habenula. cFos expression is also observed in the medial portion of the lateral habenula, an area that receives dense DA innervation via the fr and the paraventricular nucleus of the thalamus, a stress sensitive area that also receives dopaminergic input. High-intensity footshock (120, 0.8 mA shocks over 40 min) also elevates cFos expression in the rostromedial tegmental area, medial and lateral aspects of the lateral habenula and the paraventricular thalamus. In contrast to low-intensity footshock, increases in cFos expression within the rostromedial tegmental area are not altered by fr lesions suggesting a role for non-habenular inputs during exposure to highly aversive stimuli. These data confirm the involvement of the lateral habenula in modulating the activity of rostromedial tegmental area neurons in response to mild aversive stimuli and suggest that dopamine input may contribute to footshock- induced activation of cFos expression in the lateral habenula.

## Introduction

Transient changes in midbrain dopamine (DA) neuron activity appear to encode a signal that optimizes action selection by promoting rewarding actions and suppressing non-optimal behaviors [1], [2], [3]. While midbrain DA neurons receive extensive glutamatergic input [4], [5] that drives reward-induced neuronal activation [6], knowledge of the inhibitory inputs responsible for transient decreases in DA neuron activity following aversive stimuli [7], [8], [9], [10] remains limited.

The habenula (Hb), a phylogenetically conserved epithalamic structure [11], [12], is functionally and anatomically well positioned for encoding, in concert with midbrain DA neurons, the motivational value of aversive stimuli [13], [14], [15]. Activation of the lateral Hb (lHb) follows both nociception [16], [17], [18] and the absence of expected rewards [19]. In addition, habenular activity in humans correlates with negative reward prediction errors [20]. Conversely, lesions of the lHb in rats increase impulsivity [21], and sucrose- and cocaine-seeking [22], [23], behaviors normally associated with elevations in DA activity. This suggests that habenular activation acts as a ‘brake’ on midbrain DA neuronal firing.

Several laboratories have shown that direct activation of the lHb leads to widespread inhibition of DA neurons in the substantia nigra and ventral tegmental area (VTA) [19], [24], [25], potentially modulating negative error signals in the brain [15], [26]. Lesions of the fasciculus retroflexus (fr), the primary pathway conveying lHb projections to the midbrain, block lHb-induced DA inhibition [25]. However, since lHb efferents are glutamatergic [4], [27], [28], a disynaptic pathway is implied, which has been shown to include an intervening GABAergic neuron [25]. Although glutamatergic axons arising from the lHb primarily synapse on GABAergic neurons in the midbrain [27], lHb innervation of substantia nigra and VTA neurons is relatively sparse and does not selectively target GABAergic neurons [28], suggesting they are not solely responsible for the population-level inhibition of DA neuron activity observed in response to lHb stimulation.

A recently described brain region, the mesopontine rostromedial tegmental area (RMTg or tail of the VTA) receives a dense projection from the lHb, is comprised of GABAergic neurons that project massively to midbrain DA neurons [29], [30] and expresses Fos positive cells in response to psychostimulants such as d-amphetamine [31] and aversive stimuli such as footshock [32]. RMTg neurons are excited by footshock, cues predicting footshock, and the unexpected omission of a predicted reward [32], [33]. The RMTg is also strongly activated by the same stimuli that activate lHb neurons [34]. RMTg lesions in rats impair the expression of fear- and anxiety-related behaviors [32], which could reflect a deficit in the encoding of aversive stimuli. These data suggest that the RMTg is likely to be a component of a circuit encoding aspects of aversive stimuli, serving as an inhibitory relay between the lHb and midbrain DA neurons [35].

Since the lHb projects heavily to the RMTg, and both areas respond similarly to aversive stimuli, it is plausible that removal of habenular input would diminish RMTg activation following an aversive stimulus. To test this hypothesis, we quantified cFos-like immunoreactivity in the RMTg following electrolytic lesions of the fr using two footshock protocols: low-intensity shock previously shown to produce cFos expression in the RMTg [32] and high-intensity shock, predicting that fr lesions would diminish cFos expression in the RMTg of shocked rats. Since DA efferents ascend within the fr forming reciprocal connections with LHb projection neurons [36], [37], and these efferents would also be destroyed during fr lesion, we also sought to determine whether ascending projections contribute to the response of LH neurons to nociceptive stimuli. To accomplish this, cFos expression following shock was determined in each of the three principal subregions of the Hb including the medial (mHb), medial subnucleus of the lateral (lHbm), and lateral subnucleus of the lateral (lHbl) habenula. cFos expression in the posterior paraventricular nucleus of the thalamus (PVTp), a DA innervated, stress responsive structure, was also assessed.

## Materials and Methods

### Ethics Statement

This study was conducted in strict accordance with recommendations in The Guide for the Care and Use of Laboratory Animals of the National Institutes of Health [38]. All procedures were approved by the University of Maryland School of Medicine Institutional Animal Care and Use Committee (A3200-01).

### Subjects

Sixty-nine male Sprague-Dawley rats (250–270 g; Charles River, Wilmington, MA) were delivered to the animal facilities at the Maryland Psychiatric Research Center and maintained on a 12∶12 h light:dark cycle with food and water *ad libitum*. Rats were given a minimum of 48 hr to acclimate before surgery. Coordinates for lesions and structure demarcations were taken from Paxinos and Watson [39].

### Surgery

At the time of surgery, rats were anesthetized with isoflurane (2–5% in 100% O_2_) to the point of non-responsiveness to a toe pinch and maintained at that level throughout the procedure. A feedback controlled heating pad was used to maintain body temperature at 36°C. Rats were mounted in a stereotaxic instrument using atraumatic ear bars and two small burr holes were drilled through the skull above and lateral to the fr (AP: −4.5; ML: +/−2.0) before retracting the dura. Bilateral cathodal lesions of the fr were produced by passing a constant current (0.35 mA, 8s; Isolated Pulse Stimulator Model 2100, A-M Systems, Carlsborg, WA) through a concentric, bipolar electrode (SNEX-100X, Rhodes Medical Instruments, Summerland, CA) positioned within the fr (DV: –7.5 @ 10°). Sham rats underwent the same surgical procedure but no current was passed through the electrodes. All rats were given 10 days to recover before being exposed to shock.

### Shock Procedure

Sham and fr lesioned rats were randomly assigned to either shocked or unshocked groups. Three days prior to shock exposure, all rats were habituated to the shock environment for a time period equivalent to that of the shock session. The shock environment was one side of a two chamber shuttle box (21×21×16 cm; Med Associates, St. Albans, VT) configured to deliver scrambled shocks to metal floor bars. The chamber was equipped with a pair of parallel horizontal infrared photobeams positioned 3 cm above the floor and 12 cm apart. Photobeam breaks were recorded as a measure of locomotion in the high-intensity footshock experiment. Between sessions, the box was wiped clean with 70% ethanol. Experiments utilizing low- and high-intensity shock procedures were not run concurrently resulting in procedural differences that are described below.

Rats in the low-intensity footshock group received four, 0.5 mA shocks (duration 0.5 s) across a 20 minutes session. Rats in the high-intensity footshock group were exposed to a modified version of learned helplessness induction [40] receiving 120, 0.8 mA shocks (pseudo-random duration of 5 s to 15 s) across a 40 minute session. At the end of the session all rats were returned to their home cage. Ninety minutes after the start of the shock session rats were administered 0.5 ml ip Euthasol (390 mg/ml sodium pentobarbital and 50 mg/ml phenytoin sodium; Virbac Animal Health, Ft. Worth, TX) and perfused transcardially with 100 ml 4°C phospate buffered saline (PBS). This was followed by perfusion with 500 ml freshly made 4% paraformaldehyde solution, in 0.1 M phosphate buffer (PB; pH 7.4, 4°C, low-intensity footshock groups) or 500 ml 6% formalin (pH 7.4, 4°C; high-intensity footshock groups).Brains were rapidly removed and post-fixed 12 hours (low-intensity) or 30 min (high-intensity groups) prior to sectioning on a vibrating tissue slicer (VT 1200, Leica, Buffalo Grove, Il).

### Immunohistochemistry

Coronal sections (40 µm) were obtained through the rostral-caudal extent of the Hb and RMTg. Sections not processed for immediate immunostaining were stored in cryoprotectant (30% sucrose, 30% ethylene glycol, 1% PVP-40 in PBS at 4°C). For cFos staining we used the 3–3′-daminobenzidine (DAB) reaction with nickel enhancement. Omission of the primary and secondary antibodies were used as negative controls in all incubations.

### cFos with Low-intensity Footshock

Systematically random sampled sections spaced at 240 µm apart were incubated at 4°C successively, with 3 PBS rinses following each step, in: 1) 0.3% H_2_O_2_ in PBS for 30 min, 2) 3.0% Normal goat serum, 0.3% Triton-X in PBS for 2 hrs, 3) rabbit anti-cFos polyclonal primary antibody (Ab5, 1∶5000; EMD Chemicals, San Diego, CA), 1.0% Normal goat serum, 0.3% Triton-X in PBS for 60 hours, 4) biotinylated goat anti-rabbit secondary antibody (BA-1000; 1∶600; Vector Laboratories, Burlingame, CA), 1.0% Normal goat serum, 0.3% Triton-X in PBS for 2 hours, 5) avidin-biotin immunoperoxidase (ABC Elite Kit PK-6100, Vector Laboratories, Burlingame, CA) in PBS for 30 min, and 6) 0.03% DAB, 0.02% nickel ammonium sulfate in PBS for 2–5 min.

### cFos with High-intensity Footshock

The immunostaining procedure with the high-intensity footshock groups was altered to match the procedure used in other laboratories investigating cFos expression in the RMTg [32]. Systematically random sampled sections spaced at 120 µm apart were incubated at room temperature successively, with 3 PBS rinses following each step, in 1) 0.3% H_2_O_2_ in PBS for 30 min, 2) rabbit anti-cFos polyclonal primary antibody (1∶5000), 3.0% Normal goat serum, 0.3% Triton-X in PBS overnight, 3) biotinylated goat anti-rabbit secondary antibody (1∶600), 1.0% Normal goat serum, 0.3% Triton-X in PBS for 30 min, 4) avidin-biotin immunoperoxidase in PBS for 30 min, and 5) 0.03% DAB, 0.02% nickel ammonium sulfate in PBS for 2–5 min. This change in the immunostaining procedure led to increased object/background contrast relative to the procedure used in the low-intensity experiment.

### Tyrosine Hydroxylase (TH)

Sections adjacent to cFos processed sections were immunostained for TH using DAB-nickel with cobalt chloride to create a gray-blue product. Sections were incubated at room temperature successively, with 3 PBS rinses following each step, in 1) 0.3% H_2_O_2_ in PBS for 30 min, 2) mouse anti-TH monoclonal primary antibody (22941; 1∶50 000; Immunostar, Hudson, WI), 3.0% Normal goat serum, 0.3% Triton-X in PBS overnight, 3) biotinylated goat anti-mouse secondary antibody (BA-9200; 1∶400; Vector Laboratories, Burlingame, CA), 1.0% Normal goat serum, 0.3% Triton-X in PBS for 30 min, 4) avidin-biotin immunoperoxidase in PBS for 30 min, and 5) 0.03% DAB, 0.02% nickel ammonium sulfate, 0.02% cobalt chloride in PBS for 30–60 s.

### Quantification of cFos Positive Objects and TH Expression

All sections were mounted on glass slides, dried overnight, and coverslipped. For the Hb, PVTp, and RMTg, sections were analyzed that fell within –2.9 to –4.0, –2.9 to –4.0, and –6.0 to –7.0 mm from Bregma respectively. Photographs were taken of each stained section using an Axioplan microscope with DP Controller software (Olympus, Center Valley, PA) at 10 ×. Profile counts of cFos positive objects in the mHb, lHbm, lHbl, and PVTp were conducted at 20 × on the same microscope. Profile counts of cFos positive objects in the RMTg were obtained at 20 × on an BH-2 microscope with a camera lucida attachment (Olympus, Center Valley, PS). An object was counted as cFos positive if it 1) fell within the demarcated borders of the region of interest described below, 2) was between 5 and 15 µm in diameter (mean size in all areas was 9 µm), 3) had a round or ovular appearance, and 4) could easily be differentiated from the background stain.

**Figure 1:**
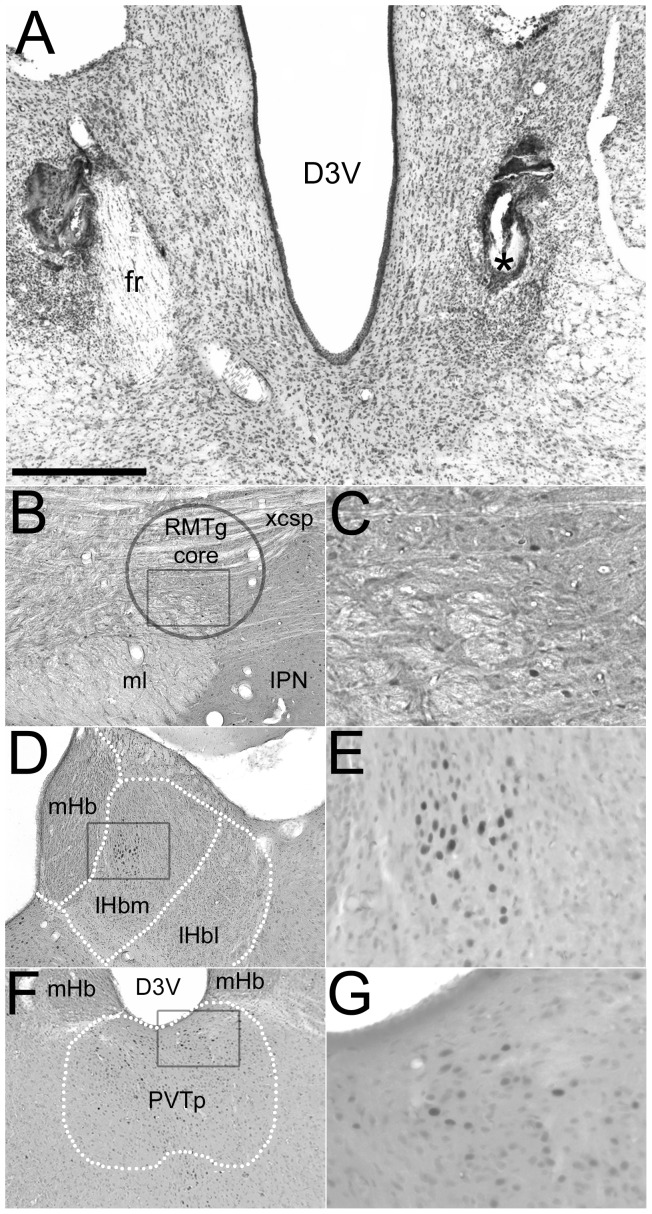
Representative photomicrographs illustrating the effects of low-intensity footshock on the expression of cFos in the RMTg, Hb and PVTp in a sham operated rat. Cresyl violet stain of a section with an incomplete fr lesion (left) and acceptable lesion (right, *) from an excluded rat (A). cFos expression within the RMTg (B,C), habenula (D,E) and PVTp (F,G). Boxes within the low-magnification micrographs (left) approximate the area of the high-magnification illustrations (right), which show visible cFos positive objects. The RMTg core is the area within the circle (B; for a complete description of RMTg boundaries see [30], [31]). Dotted lines delineate the mHb, lHbm, and lHbl (D) and PVTp (F).Scale bar = 500 µm (A, B, D, F), 125 µm (C, E, G). D3V = dorsal third ventricle, xcsp = decussation of the superior cerebellar peduncle, IPN = interpeduncular nucleus, ml = medial lemniscus,

Demarcation of the borders of the Hb subregions (mHb, lHbm, lHbl) and PVTp were outlined on photomicrographs of each structure of interest for each section using Paxinos and Watson as a guide ([39], see Figure 1D,F). Demarcation of the RMTg core and periphery was done in accordance with previous anatomical descriptions [[32],[30], see Figure 1B]. Briefly, a 500 µm diameter circle overlaid on the decussating fibers of the tegmentum and superior cerebellar peduncle constituted the RMTg core, while a 1000 µm diameter circle positioned with its center on the lateral edge of the RMTg core constituted the RMTg periphery.

Total volume of the Hb, Hb subregions (mHb, lHbm, lHbl) and PVTp was determined by using commercial software (AIS, Imaging Research Inc.) to calculate the area of each demarcated region of interest for each sampled section. These areas, the number of sections, and distance between sections were used to determine the reference volume (V_ref_) using the Cavalieri method. V_ref_ for the RMTg core and periphery was calculated in a similar manner within fixed areas demarcated by the circular borders. TH immunostaining in the Hb and PVTp was outlined and the areas of these regions used to determine TH V_ref_ in the same manner. Using counts of cFos positive objects and the V_ref_, the estimated number of cFos positive objects per mm^3^ (N_v_) was determined for each region of interest utilizing an Abercrombie correction factor with an average object size of 9 µm.

### Lesion Determination and Statistics

fr lesions from cresyl violet stained sections for each rat were drawn using the camera lucida at 10×. Area of the remaining fr was calculated bilaterally and compared to shams. Rats were included in the study if lesions reduced the fr by 50% or more on each side. Fourteen rats were excluded from the study based on suboptimal lesions (n = 9), death during the surgical recovery period (n = 2), or poor tissue preservation (n = 3). Average lesion size of rats included in the study was 72% (IQR, 62–82%). An excluded rat with one suboptimal and one acceptable lesion is shown in Figure 1A. Data were analyzed using a three-way mixed analysis of variance (ANOVA; shock×lesion×subregion) for the RMTg core/periphery and a two-way ANOVA (shock×lesion) for all other brain areas.Tukey’s test was utilized for all *post hoc* comparisons of ANOVAs with significant interaction effects. All data are expressed as the arithmetic mean ± standard error of the mean.

## Results

Representative examples of cFos expression following exposure to low-intensity footshock in a sham rat are shown in Figure 1 for the RMTg (1B and 1C), Hb (1D and 1E) and PVTp (1F and 1G; see Figure S1 for representative photomicrographs from a footshocked, fr lesioned rat). cFos expression in the Hb following low-intensity footshock appeared to be limited to TH positive areas of the lHbm. Visual inspection suggested that TH innervation of the Hb was diminished in fr lesioned rats (Figure S3). This was confirmed by comparing the volume of TH immunoreactive fibers in both low-intensity (F_(1,24)_ = 5.00, p<0.05) and high-intensity (F_(1,21)_ = 85.85, p<0.05) experiments. There was no effect of footshock on TH expression in the habenula. TH expression within the PVTp was unaffected by fr lesion in the both the low-intensity (F_(1,23)_ = 0.12, p>0.05) and high-intensity (F_(1,21)_ = 1.32, p>0.05) groups. Locomotion during the three-habituation days preceding shock exposure was unaffected by fr lesion.

### Low-intensity Footshock

**Figure 2:**
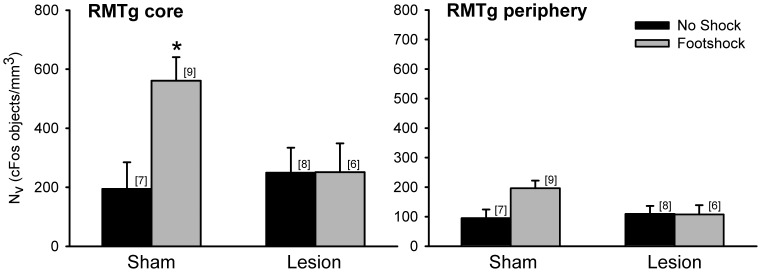
cFos positive objects in the RMTg of rats exposed to low-intensity footshock. Exposure to shock elevated cFos expression in the RMTg. Lesions of the fr prevented the increase in shock-induced cFos expression in the RMTg core; shocked sham rats showed significantly elevated cFos expression (*, Tukey, p<0.05) relative to the other three conditions. In this and in all subsequent figures the number of subjects per group are indicated above each bar.

The number of cFos positive objects within the RMTg of sham rats was increased by low-intensity footshock both in the core (+188%) and periphery (+106%) relative to unshocked shams (Figure 2). By contrast, cFos expression in fr lesioned rats exposed to low-intensity footshock was nearly identical to that of unshocked lesioned rats in both the RMTg core (+1%) and periphery (−2%). A three-way mixed ANOVA (shock×lesion×RMTg subregion) revealed a significant effect of shock (F_(1,26)_ = 4.36, p<0.05), significant difference in RMTg subregion (F_(1,26)_ = 29.96, p<0.05), and significant shock×lesion interaction (F_(1,26)_ = 4.36, p<0.05). Post-hoc analysis confirmed a significant elevation of cFos positive objects in the RMTg core of footshocked shams compared to the RMTg core of no shock shams, no shock fr lesioned rats, and footshocked fr lesioned rats (Tukey, p<0.05). Differences among groups within the RMTg periphery were not significant.

**Figure 3:**
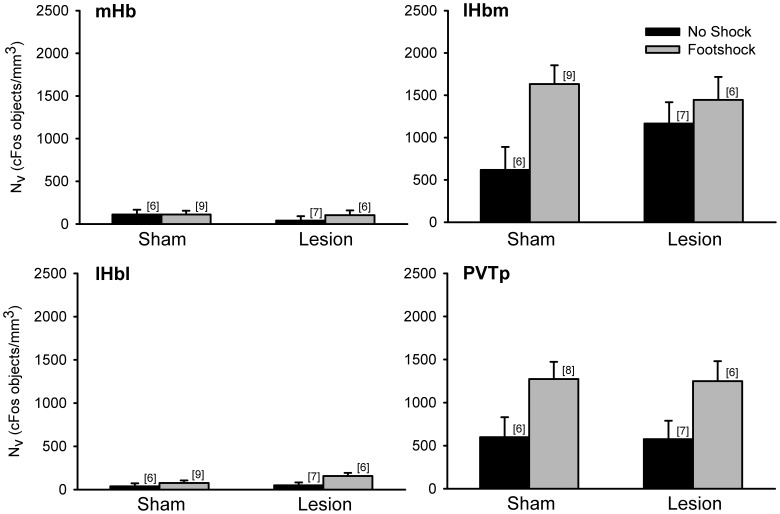
cFos positive objects in the Hb of rats exposed to low-intensity footshock. Neither shock nor fr lesions altered cFos expression in the mHb. There was a significant effect of shock for the lHbm, lHbl, and PVTp. However, there was no significant effect of lesion or shock×lesion interaction for these three areas.

Since the Hb also responds to aversive stimuli, we counted cFos positive objects in each of its three subdivisions following low-intensity footshock. Within the mHb there was no significant main effect of shock or fr lesion (Figure 3). While cFos expression within the lHbm was elevated in footshocked shams relative to no shock shams, differences between these groups were attenuated in fr lesioned rats. Indeed there was a significant main effect of shock (F_(1,24)_ = 6.40, p<0.05) but the lesion×shock interaction fell short of significance (F_(1,24)_ = 2.07, p>0.05). There was also a significant effect of shock in the lHbl (F_(1,24)_ = 4.81, p<0.05), which was likely attributable to the difference between shocked and unshocked rats in the fr lesioned group since shock appeared to have no effect within the sham group. However, for the lHbl the shock×lesion interaction was not significant (F_(1,24)_ = 1.05, p>0.05).

cFos counts within the PVTp were also obtained (Figure 3). The PVTp, like the Hb, expresses Fos in response to aversive stimuli [41], [42], [43], psychostimulants [44] and receives DA innervation from the midbrain [45], [46], [47], [48]. Analysis of cFos positive objects in the PVTp (Figure 3D) showed a significant main effect of shock (F_(1,23)_ = 9.41, p<0.05) with no effect of lesion (F_(1,23)_ = 0.01, p>0.05) or the shock×lesion interaction (F_(1,23)_ <0.01, p>0.05).

### High-intensity Footshock

**Figure 4:**
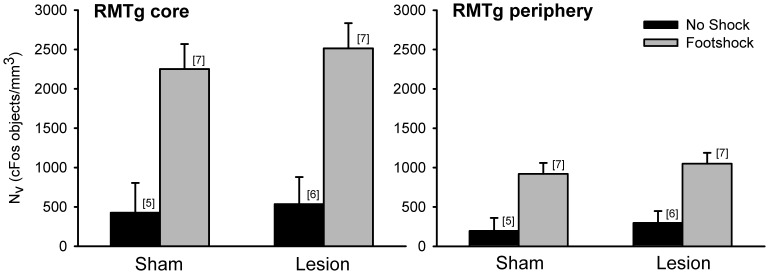
cFos positive objects in the RMTg of rats exposed to high-intensity footshock. Exposure to shock elevated cFos expression in the RMTg. Lesions of the fr had no effect on shock-induced cFos expression. Elevated cell counts in no shock rats relative to the low-intensity experiment may be the result of a change in immunostaining procedures (see Methods).

The number of cFos positive objects in the RMTg was elevated in footshocked shams relative to their no shock counterparts in both the core (+428%) and periphery (+624%; Figure 4; Figure S3). Within fr lesioned rats, shock also elevated cFos in the RMTg core (+379%) and periphery (+253%) relative to no shock fr lesioned rats (Figure S4). Overall, a three-way ANOVA (shock×lesion×RMTg subregion) revealed a significant effect of shock (F_(1,21)_ = 29.76, p<0.05), and significant difference in RMTg subregion (F_(1,21)_ = 62.59, p<0.05). In contrast to the low-intensity experiment, footshock appeared to have the same effect on cFos expression in sham and fr lesioned rats as there was no significant main effect of lesion (F_(1,21)_ = 0.390, p>0.05).

**Figure 5:**
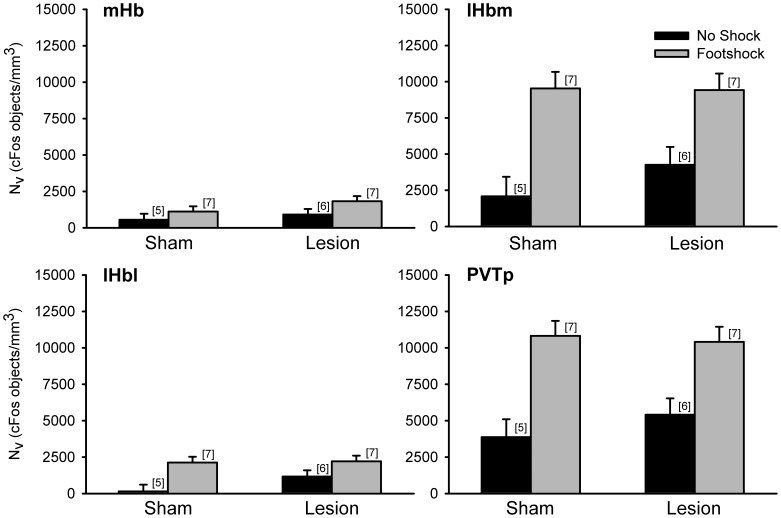
cFos positive objects in the Hb of rats exposed to high-intensity footshock. Neither shock nor fr lesion had an effect of cFos expression in the mHb. There was a significant effect of shock for the lHbm, lHbl, and PVTp. However, there was no significant effect of lesion or shock×lesion interaction for these three areas.

The effects of high-intensity footshock on cFos expression in the Hb are illustrated in Figure 5. Although there was a trend toward elevated cFos in the mHb following shock (F_(1,21)_ = 3.97, p = 0.06), no significant effects were present. Within the lHbm, there was a significant main effect of shock (F_(1,21)_ = 26.61, p<0.05) while the main effect of lesion for the lHbm was not significant (F_(1,21)_ = 0.71, p>0.05). Similarly, there was a significant main effect for shock (F_(1,21)_ = 12.91, p<0.05) in the lHbl with no significant main effect of lesion (F_(1,21)_ = 1.73, p>0.05). Like the lHbm, shock significantly increased cFos expression in the PVTp (F_(1,21)_ = 29.08, p<0.05) and there was no main effect of lesion (F_(1,21)_ = 0.26, p>0.05). There were no significant shock×lesion interactions in the high-intensity group in any of the areas examined.

## Discussion

Results from the present study provide new insights into the way aversive stimuli are processed by the habenular-RMTg circuit. First, low-intensity footshock increases cFos expression in lHb and the recently identified RMTg. Our results further show that lesions of the fr reduce footshock-induced cFos expression in the RMTg core, which implies that habenular efferents play a significant role in the activation of RMTg neurons in response to mildly aversive stimuli. However, fr lesions had no effect on RMTg cFos expression following high-intensity footshock. Since fr lesions were partially incomplete we cannot exclude the possibility that the remaining fibers in the fr were recruited during high-intensity stimulation. However, we found no evidence of a contribution made by lesion size to the main effect of shock on cFos expression in the RMTg (data not shown). The inability of fr lesions to prevent cFos expression in the RMTg following high intensity footshock suggests that other inputs to this region mediate this effect, possibly including brain areas directly involved in nociception. While Hb neurons exhibit graded responses to peripheral noxious stimuli there are no direct sensory nociceptive inputs to the lHb and lesions of the structure fail to alter pain threshold [49] suggesting that habenular neurons are not directly involved in encoding pain intensity. Rather, lHb neurons appear to receive a “copy” of nociceptive information for use in behavioral integration [50]. It is possible that inputs to the RMTg from the Hb are among the first to be activated in response to mildly aversive stimuli. However, as the aversive nature of the stimulus increase, other afferent inputs to the RMTg become principally responsible for driving the observed changes in cFos expression. It is worth noting in this regard that the RMTg receives one of its strongest inputs from the ventrolateral periaqueductal gray (PAG), a brain area that receives direct input from the spinothalamic tract and serves as a key component in nociception [29], [30].

Second, while confirming that the habenula shows elevated cFos expression in response to aversive stimuli [51], [52] we have also shown that these changes are regionally specific. The mHb appears to be completely unaffected by low- or high-intensity footshock, while the lHb responded to both low- and high-intensity footshock. cFos elevation occurs largely in the medial portion of the lHb a finding previously shown with novel and aversive stimuli [51]. Elevated cFos expression in the lateral portion of the lHb has been repeatedly observed in response to psychomotor stimulants such as amphetamines [51], [53], [54], and cocaine [55], [56]. It is worth noting that in the present work high-intensity footshock, like psychomotor stimulants, resulted in a substantial increase in cFos expression in the lHbl and that the lHbl receives a strong projection from the internal segment of the globus pallidus [57], an area critically involved in the regulation of voluntary movement. While we found no difference in locomotion between shocked and unshocked rats, the photobeams used here were not sensitive to vertical movement and consequently may not adequately account for all aspects of movement. Further studies would be necessary to test whether elevated lHbl cFos expression following footshock is a result of changes in locomotion.

Third, fr lesions may alter cFos expression in the Hb itself, as suggested by the trend toward an increase in cFos expression in the lHbm of fr lesioned rats not exposed to footshock. Moreover, as shown in Figure 1D and 1E, the increase in cFos expression following low-intensity footshock is localized in the lHbm near the parvocellular subnucleus. The lHbm contains a high-density of TH positive fibers (Figure S2A and [58]) and DA concentrations in this subregion are dramatically reduced by 6-OHDA lesions of the VTA [59]. Since ascending DA projections to the Hb travel within the fr [36], [37], lesions of this pathway would be expected to reduce DA innervation of the Hb. This supposition is supported by the decrease in habenular TH immunostaining following fr lesion observed in the present study. While the effects of DA on lHb neurons are incompletely understood, the habenula shows decreased glucose utilization following acute administration of direct and indirect DA agonists [60], [61] while showing increased utilization following acute administration of DA antagonists [61] and during morphine withdrawal [62]. These data suggest that DA in the lHbm is inhibitory and potentially involved in regulating the habenular response to aversive stimuli. Supporting this proposition, single pulse stimulation of the VTA leads to transient inhibition of pain responsive neurons in the LHb [63]. Given the tonic nature of DA neuron activity in vivo, loss of DA innervation of the lHbm may increase the basal activity of these habenular neurons via disinhibition (see however [64]). Such an increase in basal activity may explain why fr lesioned, unshocked rats had a trend, though non-significant, toward increased cFos expression within the lHbm. In support of this premise, tyrosine depletion reduces amphetamine-induced Fos immunoreactivity in the lHb of rats [65].

There are a number of alternate possibilities to consider. For example, it is conceivable that lesioning the descending fibers of the fr leads to retrograde neural degeneration and non-specific cFos expression in the Hb. Given that habenular efferents traveling within the fr originate from all regions of the nucleus it would be expected that fr lesions would increase cFos expression throughout the Hb. However, fr lesions failed to increase cFos expression in the mHb or lHbl of unshocked rats (Figures 3 and 5). Alternatively, fr lesions could lead to a generalized disruption in the encoding of aversive stimuli. To test this, we assessed cFos expression in the PVTp, an area in close proximity to the habenula that receives DA input [44], [45], and is also responsive to aversive stimuli [41], [42], [43]. Despite these similarities to the lHbm we did not see a similar pattern of change in PVTp cFos expression. While generally supportive of a role for DA in altering habenular activity during exposure to aversive stimuli further studies are needed to clarify this feedback mechanism.

Recent tract tracing studies have shown that habenular projections to the RMTg arise mainly in the lHbl while the LHbm contributes to a lesser extent [66]. These authors also demonstrated that habenular projections to the VTA, specifically the DA neuron dense paranigral subnucleus, arise mainly from the parvocellular subnucleus of the lHbm. This would suggest that aversive events that activate the lHbm cause direct activation of some DA neurons. While the firing rate of most midbrain DA neurons are inhibited by aversive stimuli [10], a minority are activated by these events [67], [68], [69], [70], and by stimuli predictive of them [68], [69], [70]. Thus, direct glutamatergic projections from the lHbm to the substantia nigra and VTA could account for those DA neurons activated by aversive stimuli while feedforward inhibition mediated by lHb-induced activation of RMTg neurons is likely to account for the predominant inhibition seen after aversive stimuli.

Our current data demonstrate that the habenula plays a role in regulating RMTg activation following mildly aversive stimuli, habenular activation following aversive stimuli is sub-region specific, and may be altered by the loss of DA input. Since the habenula is affected by other monoamines [71], [72] it will be important to ascertain to what extent changes seen here may be due specifically to the loss of DA input. While our results are consistent with the involvement of other afferents in activating RMTg neurons in response to noxious stimuli, they demonstrate that the habenula-RMTg pathway plays a role in the processing of mild aversive stimuli.

## Supporting Information

Figure S1 **Representative photomicrographs illustrating the effects of low-intensity footshock on the expression of cFos in the RMTg, Hb and PVTp in a fr lesioned rat.** cFos expression within the RMTg (A,B), habenula (C,D) and PVTp (E,F). Boxes within the low-magnification micrographs (left) approximate the area of the high-magnification illustrations (right), which show visible cFos positive objects within the Hb and PVTp, but not within the RMTg core. The RMTg core is the area within the circle (A). Dotted lines delineate the mHb, lHbm, and lHbl (C) and PVTp (E).Scale bar = 500 µm (A,C,E), 125 µm (B,D,F).
(TIF)

Figure S2 **Representative photomicrographs illustrating the effects of fr lesion on the expression of TH in the Hb and PVTp.** TH immunostaining (dark grey) in the Hb of a sham (A) and lesioned (B) rat Illustrates the significant decrease in habenular TH expression following fr lesion. TH immunostaining in the PVTp is unaffected by fr lesion, as illustrated by comparing a sham (C) and fr lesioned rat (D). Scale bar = 500 µm.
(TIF)

Figure S3 **Representative photomicrographs illustrating the effects of high-intensity footshock on the expression of cFos in the RMTg, Hb and PVTp in a sham operated rat.** cFos expression within the RMTg (A,B), habenula (C,D) and PVTp (E,F). Boxes within the low-magnification micrographs (left) approximate the area of the high-magnification illustrations (right), which show visible cFos positive objects. The RMTg core is the area within the circle (A). Dotted lines delineate the mHb, lHbm, and lHbl (C) and PVTp (E).Scale bar = 500 µm (A,C,E), 125 µm (B,D,F).
(TIF)

Figure S4 **Representative photomicrographs illustrating the effects of high-intensity footshock on the expression of cFos in the RMTg, habenula and PVTp in an fr lesioned rat.** cFos expression within the RMTg (A,B), habenula (C,D) and PVTp (E,F). Boxes within the low-magnification micrographs (left) approximate the area of the high-magnification illustrations (right), which show visible cFos positive objects. The RMTg core is the area within the circle (A). Dotted lines delineate the mHb, lHbm, and lHbl (C) and PVTp (E).Scale bar = 500 µm (A,C,E), 125 µm (B,D,F).
(TIF)
