# Supplementary figures and images for: Lesions of the Fasciculus Retroflexus Alter Footshock-Induced cFos Expression in the Mesopontine Rostromedial Tegmental Area of Rats
Source: PLoS One. 2013 Apr 12;8(4):e60678. doi: 10.1371/journal.pone.0060678 (PMC3625179; doi:10.1371/journal.pone.0060678)

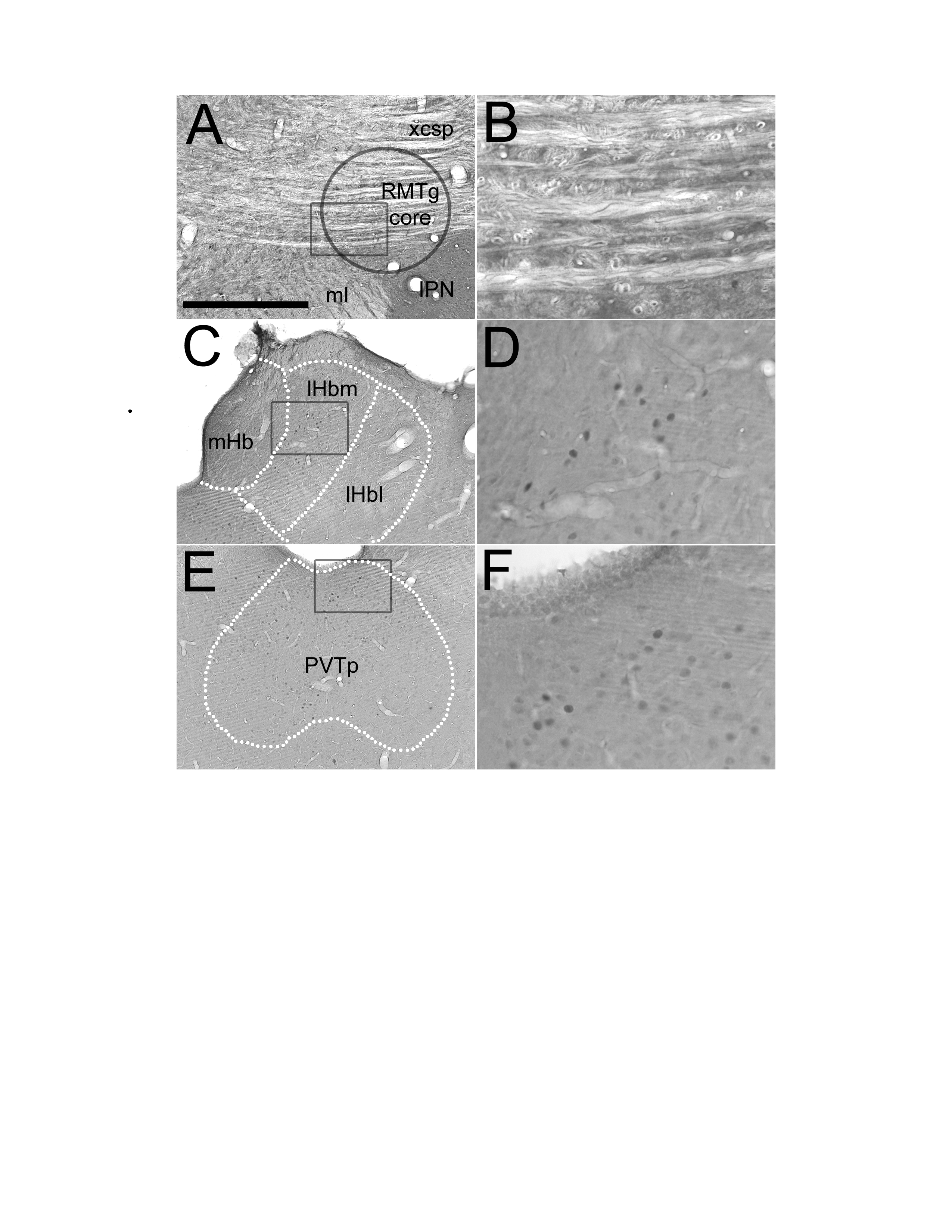

Supplement: Figure S1 — Representative photomicrographs illustrating the effects of low-intensity footshock on the expression of cFos in the RMTg, Hb and PVTp in a fr lesioned rat. cFos expression within the RMTg (A,B), habenula (C,D) and PVTp (E,F). Boxes within the low-magnification micrographs (left) approximate the area of the high-magnification illustrations (right), which show visible cFos positive objects within the Hb and PVTp, but not within the RMTg core. The RMTg core is the area within the circle (A). Dotted lines delineate the mHb, lHbm, and lHbl (C) and PVTp (E).Scale bar = 500 µm (A,C,E), 125 µm (B,D,F). (TIF) [file pone.0060678.s001.tif]

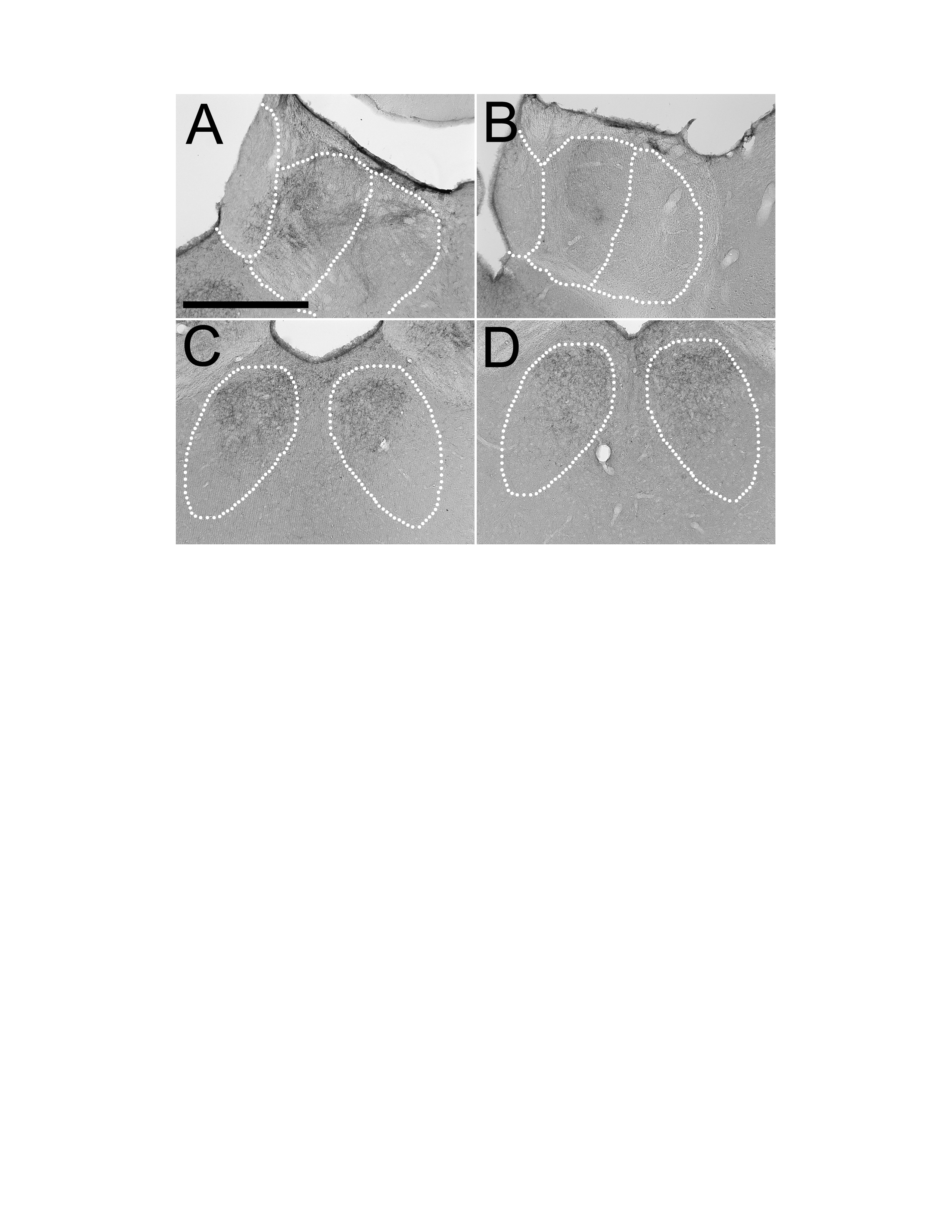

Supplement: Figure S2 — Representative photomicrographs illustrating the effects of fr lesion on the expression of TH in the Hb and PVTp. TH immunostaining (dark grey) in the Hb of a sham (A) and lesioned (B) rat Illustrates the significant decrease in habenular TH expression following fr lesion. TH immunostaining in the PVTp is unaffected by fr lesion, as illustrated by comparing a sham (C) and fr lesioned rat (D). Scale bar = 500 µm. (TIF) [file pone.0060678.s002.tif]

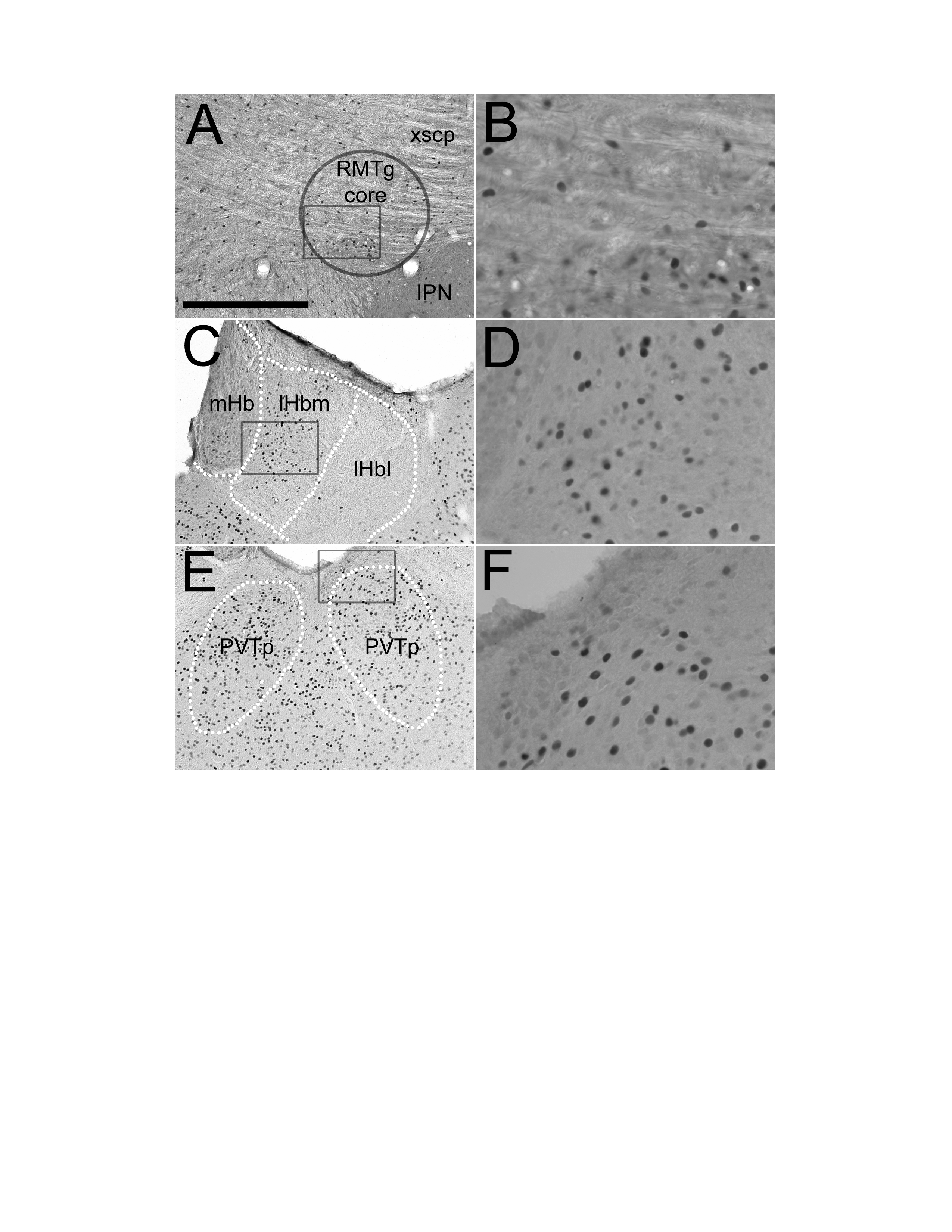

Supplement: Figure S3 — Representative photomicrographs illustrating the effects of high-intensity footshock on the expression of cFos in the RMTg, Hb and PVTp in a sham operated rat. cFos expression within the RMTg (A,B), habenula (C,D) and PVTp (E,F). Boxes within the low-magnification micrographs (left) approximate the area of the high-magnification illustrations (right), which show visible cFos positive objects. The RMTg core is the area within the circle (A). Dotted lines delineate the mHb, lHbm, and lHbl (C) and PVTp (E).Scale bar = 500 µm (A,C,E), 125 µm (B,D,F). (TIF) [file pone.0060678.s003.tif]

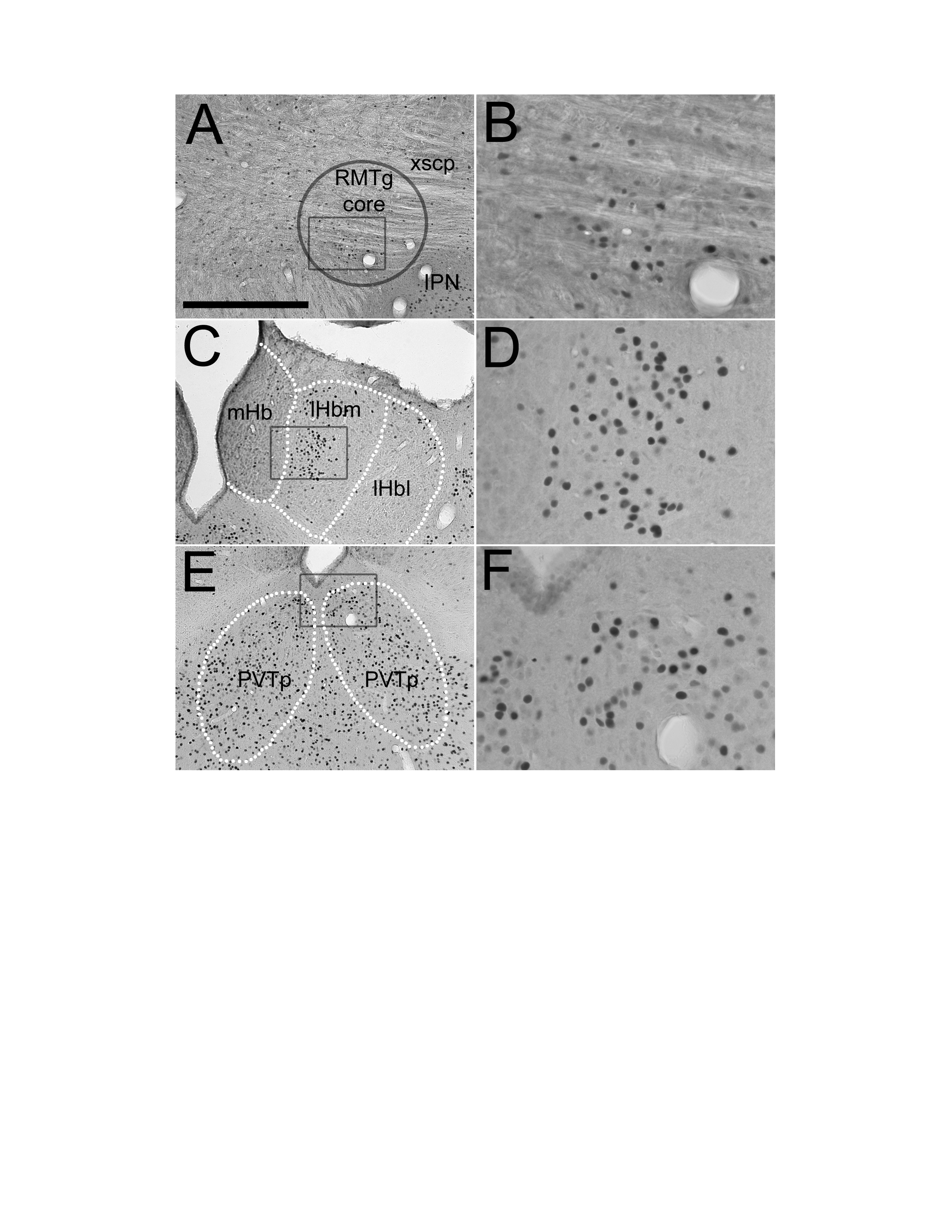

Supplement: Figure S4 — Representative photomicrographs illustrating the effects of high-intensity footshock on the expression of cFos in the RMTg, habenula and PVTp in an fr lesioned rat. cFos expression within the RMTg (A,B), habenula (C,D) and PVTp (E,F). Boxes within the low-magnification micrographs (left) approximate the area of the high-magnification illustrations (right), which show visible cFos positive objects. The RMTg core is the area within the circle (A). Dotted lines delineate the mHb, lHbm, and lHbl (C) and PVTp (E).Scale bar = 500 µm (A,C,E), 125 µm (B,D,F). (TIF) [file pone.0060678.s004.tif]
